# IL-1β in Neoplastic Disease and the Role of Its Tumor-Derived Form in the Progression and Treatment of Metastatic Prostate Cancer

**DOI:** 10.3390/cancers17020290

**Published:** 2025-01-17

**Authors:** Yetunde Oyende, Luke J. Taus, Alessandro Fatatis

**Affiliations:** 1 Department of Pharmacology and Physiology, Drexel University College of Medicine, Philadelphia, PA 19102, USA; yeo23@drexel.edu (Y.O.); ljt39@drexel.edu (L.J.T.); 2 Sidney Kimmel Comprehensive Cancer Center, Thomas Jefferson University, Philadelphia, PA 19107, USA

**Keywords:** cytokines, metastasis, androgen deprivation therapies, androgen receptor

## Abstract

Since its discovery, IL-1β has taken center stage as a key mediator of a very broad spectrum of diseases revolving around immuno-mediated and inflammatory events. Predictably, the pleiotropic nature of this cytokine in human pathology has led to the development of targeted therapeutics with multiple treatment indications in the clinic. Following the accumulated findings of IL-1β’s central modulatory role in the immune system and the implication of inflammatory pathways in cancer, the use of IL-1β antagonists was first proposed and then also pursued for oncology disorders. However, this approach has consistently relied on the perceived need of interfering with IL-1β synthesized and secreted by immune cells. Herein, we discuss the importance of IL-1β derived from cancer cells which impacts primary tumors, particularly metastatic lesions, separately from and in addition to its more recognized role in immune-mediated inflammatory events. To this end, we focus on the instrumental contribution of IL-1β in the establishment and progression of advanced prostate adenocarcinoma. Special emphasis is placed on the potential role that the standard-of-care treatment strategies for prostate cancer patients have in unleashing IL-1β expression and production at metastatic sites. We conclude by reviewing the therapeutics currently used for blocking IL-1β signaling and propose a rationale for their concomitant use with standard-of-care treatments to improve the clinical outcomes of advanced prostate cancer.

## Simple Summary

Standard-of-care treatments for patients with metastatic prostate cancer aim to block the activation and/or signaling of the androgen receptor (AR). Although initially highly beneficial, this strategy eventually fails to control clinical progression. There is now evidence that targeting the AR causes cancer cells to secrete IL-1β, an inflammatory molecule with a recognized role in promoting the growth and survival of cancer cells in metastatic tumors. Here we propose to combine drugs that reduce plasma levels of testosterone and/or inhibit AR signaling with FDA-approved agents interfering with IL-1β signaling. We conclude by reviewing these agents and providing a rationale for their use in the clinic.

## 1. Introduction

Several excellent and comprehensive reviews have described the discovery, characterization, and pro-tumoral and anti-tumoral effects of IL-1β, combined with the rationale for each group of effects exerted by this cytokine and the therapeutic value of strategies either promoting or antagonizing IL-1β activity in patients.

This review offers a general perspective on the implication of IL-1β in neoplastic diseases and mainly focuses on the role played by this cytokine in prostate cancer and particularly on the establishment and progression of skeletal metastases in the advanced form of the disease. With this main objective in mind, we start by discussing the pro-tumoral properties of IL-1β produced and secreted by immune cells and promoting the growth of primary and metastatic tumors. Increasing and convincing evidence shows that IL-1β can also be expressed by cancer cells, indicating a tumor-autonomous role in fueling neoplastic progression, either together with or independently of the immune system. However, these newer findings demand further studies to understand how neoplastic cells execute a task commonly attributed to immune cells, which are appropriately equipped to respond to specific stimuli by recruiting downstream signaling pathways activating inflammasomes. In this context, it is also reasonable to ask whether cancer cells are endowed with constitutively active inflammasome activity and/or utilize alternate mechanisms for IL-1β transcription, cleavage of its pro-form, and secretion of the fully active form in the extracellular space. This is particularly pertinent to patients with advanced prostate cancer, who are currently treated with hormone-deprivation therapies (ADT) and/or drugs interfering with either the transcriptional activity of the androgen receptor (AR), referred to as (ARIs), or the synthesis of androgens. We will discuss findings from our group indicating that blocking AR-mediated signaling—in addition to its highly beneficial, albeit short-lived effects—can also relieve the transcriptionally repressive effects of the AR on the IL-1β gene, possibly unleashing the synthesis of this cytokine and its release in the metastatic niche.

We conclude by reviewing the results from selected clinical trials and summarize the therapeutics currently used to interfere with IL-1β activity, proposing their use in combination with the current standards of care in patients with aggressive or metastatic prostate adenocarcinoma.

## 2. IL-1β Processing, Known Receptors, and Their Signaling

IL-1β is a pro-inflammatory cytokine that belongs to the IL-1 family. In addition to IL-1β, the IL-1 family comprises other members with agonist and antagonist activities [1]. Throughout the text, we will define individual members of the IL-1 family (for example IL-1β), if their functional and/or regulatory features differ from other members and will use IL-1 when discussing features shared by or affecting all members of this family.

IL-1β is routinely synthesized during inflammation and injury by immune cells such as monocytes and macrophages [2,3,4]. In these cells, the process is started by the recruitment of the inflammasomes [5,6], a system of tightly regulated effectors of which one of the most studied is NLRP3 [7]. The resulting cascade of cellular and signaling events leads to the activation of caspase-1, a protease that cleaves the precursor of IL-1β (pro-form) into its active form, which is then secreted into the extracellular space. Notably, several studies have reported that other caspases—dependent or independent of the inflammasome components—as well as proteases not belonging to the caspase family can cleave IL-1β into its active form [8,9,10,11].

The IL-1 receptor type-1 and 2 (IL-1R1 and IL-1R2) and Toll-like receptors (TLRs) are membrane-bound receptors that play a critical role in responding to infections and cell injury [12,13]. IL-1R1 and TLR are often referred to as alarm receptors because they can be activated by pathogen-associated molecular patterns (PAMPs) and danger-associated molecular patterns (DAMPs) [14,15]. TLR and IL-1R1 initiate downstream signaling by involving the adaptor protein MyD88, among others. MyD88 oligomerizes and recruits the IL-1R-associated kinases (IRAKs) to the death domain on the receptor [13,16,17,18]. Upon binding, IRAK is activated via autophosphorylation and recruits intracellular signaling, such as that relying on the NF-κB pathway (see below). The activation of IRAK promotes the recruitment of the inflammasome components and cleavage/activation of caspase-1, leading to the synthesis and secretion of IL-1β and other inflammatory cytokines such as IL-6. Notably, upon secretion, the active form of IL-1β can bind to membrane-bound IL-1R and TLR to further stimulate its production via an autocrine/paracrine positive feedback loop [19,20,21].

Activation and signaling of the IL-1R and TLR are tightly regulated both extracellularly and intracellularly, since dysregulation of these receptors leads to diseases such as cancer, in which overt and continued inflammation promotes tumorigenesis. A mechanism of extracellular regulation of the receptors is provided by the binding of the IL-1R antagonist (IL-1Ra) [22]. IL-1Ra competes with both IL-1β and IL-1α for the IL-1R and inhibits IL-1R activation. An additional mechanism of extracellular regulation is the binding of IL-1β and IL-1α to IL-R2, which is a decoy receptor lacking a signaling domain but able to reduce the availability of both ligands for IL-1R and downstream activation [23,24,25]. Intracellular regulation of IL-1R and TLR activation is based on the intervention of the splice variants of MyD88 and IRAK-M [26,27,28].

As mentioned above, the cascade of events triggered by the stimulation of IL-1R and leading to IL-1β production is considered heavily dependent on the involvement of NF-κB signaling. NF-κB is a transcription factor involved in downstream inflammatory response. In its inactivated form, it is sequestered in the cytosol by binding to inhibitory proteins such as IκBs. Phosphorylation and degradation of the IκB proteins result in the translocation of NF-κB to the nucleus, which can modulate gene transcription [29]. Upon recognizing pathogens and activating PAMPs and DAMPs, NF-κB is activated, translocated to the nucleus, and promotes the transcription of inflammation-related genes, including the inflammasome components and pro-inflammatory cytokines (e.g., IL-1β).

Taken together, these findings lend support to the notion that activated NF-κB promotes the synthesis of IL-1β and IL-1 signaling can induce the activation of NF-κB in cells. Interestingly, Greten et al. reported an unexpected inhibitory role of NF-κB on IL-1β signaling. Genetic or prolonged pharmacological inhibition of IKKβ, a key activator of NF-κB, resulted in an increased IL-1β secretion in macrophages and neutrophils [30]. This study also reported that pharmacological inhibition or knockout of IKKβ downregulates NF-κB related genes, including pro-IL-1β levels, while increasing the secretion of the mature, active form of this cytokine. Furthermore, the increase in IL-1β secretion was reportedly due to enhanced secretion of activated caspase-1 in macrophages and serine protease activity in neutrophils [30]. Thus, cross-talks between NF-κB activation and IL-1β signaling is not a universal positive feedback mechanism but a context-dependent modulatory network.

## 3. Immune Cell-Derived IL-1β and Its Tumor-Promoting Role

Tumor immunity is a complex and dynamic process that involves the intricate interplay between cancer cells and the immune system. Among a diverse array of immune cells, several key players have been identified to exert crucial roles in recognizing, targeting, and eliminating tumor cells. Immune cells involved in tumor immunity include both T and B lymphocytes, natural killer (NK) cells, macrophages, myeloid-derived suppressor cells (MDSCs), and dendritic cells [31,32,33,34,35]. IL-1β generated by immune cells plays a major role in orchestrating immune responses and inflammation. This cytokine can suppress tumor development and progression, depending on the immune cells of origin, cancer type, and tumor stage [36,37].

On the other hand, a tumor-promoting role of IL-1β has been also well established over the years, as discussed here. The tumor microenvironment (TME) serves as a niche, promoting spatial and functional interactions between cancer cells, resident cells of the surrounding stroma, and autochthonous or migrated immune cells. Cytokines are among the soluble factors promoting these interactions and supporting tumor growth and progression. Single-cell RNA-seq datasets from patients and pre-clinical mouse models revealed that myeloid cells have the highest expression of IL-1β in breast and lung cancer [38]. The IL-1β secreted by the myeloid cells promotes lung and breast cancer progression and metastasis, combined with a decrease in immunosuppressive effects in the TME [38,39]. Furthermore, IL-1β has been detected in co-cultures of human breast cancer cells and monocytes, favoring in vitro invasion and aggressiveness [40]. These findings can be explained by the reported stimulation of IL-1β secretion from monocytes and macrophages elicited by breast cancer cells [41]. Interestingly, further pre-clinical studies showed that the absence of local macrophages reduced the incidence of metastasis to the lungs [41]. Consistently, a different study established the role of inflammasome activation in tumor-associated macrophages (TAMs) and consequent IL-1β production, which generated an inflammatory microenvironment promoting breast cancer progression [42]. Taken together, these findings highlight the importance of IL-1β produced by the immune cells in tumor progression, as further discussed in the following sections.

### 3.1. Angiogenesis and Vascularization

The formation of new blood vessels from existing vasculature and their organization into new vascular networks are essential contributors to tumor progression and metastasis, and vascular endothelial growth factor (VEGF) is a crucial player in both events. Tumor-associated macrophages in a murine model of Dalton’s lymphoma had increased IL-1β activity, measured as thymocyte proliferation, as compared to peritoneal macrophages in tumor-free animals [43]. Consistently, the stimulation of macrophages with lipopolysaccharide (LPS) under hypoxic conditions, which is often detected in both primary and secondary tumors [44,45], increases their expression of IL-1β and VEGFR, thus enabling angiogenesis [46]. Interestingly, IL-1β produced by macrophages can signal in an autocrine manner to stimulate the activation of STAT3 and NF-kB to increase the expression of VEGF-A transcripts [47]. An additional study showed that depletion of IL-1β resulted in tumor regression and reduction in tumor-infiltrating neutrophils, promoting an immunosuppressive TME and resistance to angiogenic therapy [38].

In TAMs, the activation of NLRP3 is promoted by Bruton’s tyrosine kinase (BTK), leading to IL-1β secretion in the TME [48]. Upon activation and maturation, dendritic cells can also secrete IL-1β by interacting specifically with alloreactive T cells [49]. Several other studies have also demonstrated that both the secretion and the release of IL-1β by dendritic cells depend on their interaction with T cells [4,50]. Finally, by secreting IL-1β, dendritic cells can promote angiogenesis via VEGF-A and β-defensins [47,51].

### 3.2. Immune Evasion

Multiple immune cells present in the TME, including MDSCs and Treg cells, are able to organize an immune response to kill tumor cells. However, the presence of cytokines can help surmount the activities of immune cells and instead promote immune evasion. IL-1β in the TME can result in an immunosuppressive environment that supports tumor proliferation and metastasis. Exogenous expression and secretion of IL-1β in a pre-clinical breast cancer model resulted in the recruitment and accumulation of myeloid suppressor cells, as well as the repression of CD8+ T cells [52], thus impairing their anti-cancer immune response. Consistently, pre-clinical models of breast cancer showed that blockade of IL-1β secreted by macrophages in the TME resulted in the accumulation of tumor-infiltrating dendritic cells and the tumor-infiltrating CD8+ T cells [39], promoting tumor regression over time. In addition, the absence of IL-1β in the TME delayed the differentiation of CCR2+ cells into immunosuppressive macrophages [39]. Notably, the depletion of macrophages and CD8+ T cells reverted the regression of tumors and inhibited the infiltration and expansion of neutrophils in the TME [38] of breast and lung cancer IL-1β-deficient animal models. Furthermore, CD8+ cytotoxic T cells are required to inhibit tumor progression and are critical drivers of CD4+ T cell and TAM activation in IL-1β-deficient mice [38].

## 4. Synthesis and Secretion of IL-1β by Tumor Cells

Studies conducted over the years have reported an increased expression of IL-1β in the serum of advanced cancer patients compared to samples from healthy donors [53,54,55]. Elevated IL-1β serum levels are strongly correlated with disease progression, highlighting the clinical relevance of IL-1β as a potential prognostic marker in neoplastic disease. Although these findings could result from IL-1β secretion by immune cells, as discussed above, earlier studies reported the expression of IL-1β by melanoma cells measured by PCR [56]. Several other studies have successively confirmed the expression and in vitro secretion of the IL-1β protein by melanoma cells [57] and expanded these initial observations to breast cancer cells [58,59,60]. For prostate adenocarcinoma, findings from an early immunohistochemistry study by Ricote et al. [61] were successively confirmed and extended by our group; we reported the pro-metastatic role of IL-1β derived from human prostate cancer cells and the effects of blocking IL-1β signaling on the metastatic progression in pre-clinical animal models [62,63]. Notably, these studies were also the first to report the inverse correlation between expression of IL-1β and activity of the androgen receptor [62], a crucial observation that will be discussed more in depth later. More recently, tumor-derived IL-1β was implicated in cross-talk between prostate cancer cells and adipocytes, leading to reduced sensitivity to docetaxel via lipolysis-dependent mechanisms [64].

As discussed above, immune cells commonly synthesize IL-1β in response to pathogens or sterile perturbations such as trauma, ischemia, and metabolic variations. Since tumor cells are not involved in the response to pathogens or other perturbations of the general homeostasis, their synthesis of IL-1β is a less obvious and expected event than for immune cells. Interestingly, the inflammasome cascade can also be primed by an intracellular increase in reactive oxygen species (ROS), following the activation of the extracellular signal-regulated kinase 1 (ERK1) or upon recruitment of the kinase associated with the IL-1R (IRAK1) [65]. Indeed, some studies have shown that advanced melanoma cells express constitutively active inflammasome components [57,66,67]. Similarly, other cancer types have also been reported to express high levels of NLRP3 and to secrete IL-1β, which strongly correlates with disease progression [68,69,70]. Furthermore, Beaupre et al. have proposed that the endogenous synthesis of IL-1β by leukemic cells is due to the presence and activity of Ras mutations [71]. To support their studies, they found that interfering with Ras signaling affected the production of IL-1β and the growth of leukemia cells in vitro [71]. Additional priming mechanisms may include the activation of receptor-interacting protein 1 (RIPK1) or the NF-κB pathway by radiation therapy, both leading to the production and secretion of IL-1β in lung cancer cell lines [72]. RIPK1 can form a complex with RIPK3 and Fas-associated death domain protein (FADD), which activates caspase 8, resulting in the cleavage of pro IL-1β [73,74,75]. NF-κB can be also activated by growth factors, stress inducers, and pro-inflammatory cytokines, and the activation of PI3K/Akt signaling pathway leading to an IL-1β/NF-κB positive feedback loop has been reported in hepatocytes [76]. A similar mechanism can be induced in breast cancer cells upon the activation of EGFR by amphiregulin [21], whereas autocrine and paracrine stimulation of IL-1R by IL-1β secreted by several types of tumor cells can lead to the constitutive activation of the NF-κB pathway [77]. This outcome relies on the formation of a transient complex between tumor necrosis factor receptor-associated factor 6 (TRAF6), transforming growth factor-β-activated kinase 1 (TAK1), and mitogen-activated protein kinase (MAPK) kinase 3 (MEKK3), which in turn results in the phosphorylation of IκB and the subsequent activation of NF-κB [78]. To this point, the constitutive activation of NF-κB and the expression of the E3-ubiquitin ligase receptor subunit βTRCP1 promotes the autocrine secretion of IL-1β in pancreatic cancer cell lines [79].

In support of the implication of the PI3K/Akt pathway in IL-1β signaling, the inhibition of Akt phosphorylation in lung cancer cells suppresses the activity of this cytokine and blocks NF-κB translocation into the nucleus [80]. Consistently, loss or inhibition of PTEN, an enzyme responsible for the dephosphorylation and inactivation of Akt, increases NF-κB transcriptional activity and IL-1β production in pancreatic cancer cells [81]. Interestingly, loss of PTEN and constitutive activation of NF-κB have been implicated in advanced prostate cancer [82,83]. Dysregulation of PI3K/Akt signaling and constitutively active NF-κB have been recently implicated in the upregulation of IL-1β in prostate cancer cells [84] and could be plausibly involved—at least in part—in the production and secretion of this cytokine, as previously reported for these cells [62,63].

In summary, tumor cells produce and secrete IL-1β either in conjunction with or as an alternative to immune cells, contributing to an inflammatory microenvironment but also altering the gene expression profiles of tumor-associated stromal cells, as reported in pre-clinical models of metastatic prostate cancer [63]. Notably, while the NLRP3 inflammasome was found to be responsible for IL-1β processing in pancreatic cancer cells [85], its expression and activation are mostly detected in tumor-associated stromal or immune cells rather than cancer cells [86]. In fact, the molecular mediators and intracellular pathways responsible for IL-1β production in cancer cells are still poorly defined, and their identification will likely provide additional therapeutic targets to counteract the tumor cell-intrinsic contribution to disease progression by IL-1β signaling.

## 5. Tumor-Derived IL-1β in Different Metastatic Tumors

Dissemination of tumors from their primary sites to distant organs is a major cause of patients’ demise. This section will present evidence that different tumors (prostate cancer is discussed separately below) secrete IL-1β to aid their spreading to and colonization of secondary target tissues. Metastasis is a complex process, involving epithelial–mesenchymal transition (EMT) and angiogenesis, local invasion into the systemic blood circulation, evasion of cell death by anoikis, extravasation from the blood stream, seeding into secondary organs, and finally survival and colonization at secondary sites [87,88]. Cytokines, including IL-1β and chemokines, play an essential role in metastasis. For instance, exogenous IL-1β increases the incidence of spontaneous lung metastasis in melanoma and renal carcinoma cancer cells, and treatment with IL1Ra reduces the incidence of metastasis and tumor growth in vivo [89,90,91].

### 5.1. Epithelial–Mesenchymal Transition

Epithelial–mesenchymal transition (EMT) is classically defined as the switch from an epithelial phenotype to a mesenchymal phenotype, as underscored by the decrease in epithelial markers such as E-cadherin and the increase in mesenchymal markers such as vimentin [92,93]. Over the years, several studies have demonstrated the importance of EMT in cancer cells as a critical regulator of tumor progression and its dissemination to secondary organs [93,94]. Chronic inflammation is a known regulator of EMT, including the presence of cytokines like IL-1β in cancer [95,96,97,98]. To facilitate metastasis, IL-1β can dysregulate the activation of signaling pathways and promote EMT in cancer cells. For instance, breast cancer cell lines overexpressing IL-1β showed decreased epithelial markers (E-cadherin) and increased mesenchymal markers (N-cadherin) compared to their wild-type cell lines [60]. Additionally, IL-1β stimulation promotes nuclear translocation of β-catenin and increases the transcription activities of β-catenin target genes in breast cancer via EMT induction [99]. Furthermore, Jimenez-Garduno et al. reported that IL-1β induces the hemi-methylation of the ESR1 promoter that is responsible for the downregulation of the ERα gene and the induction of EMT, as demonstrated by an increased expression of TWIST1 and the activation of the PI3K/AKT signaling pathway in TNBC in vitro models [100]. IL-1β upregulates the expression of HIF-1α, which results in the induction of EMT in hepatocellular carcinoma in vitro and in vivo [101]. Hypoxia, which can be influenced by IL-1β expression [102,103], was also reported to induce EMT in colorectal cancer [104]. Thus, the presence of IL-1β can promote tumor progression, migration, and metastases by inducing EMT in cancer.

### 5.2. Invasion

Cancer cell invasion is characterized by a breach of the basement membrane, resulting in the spreading of cancer cells from their primary site to lymph nodes and into the bloodstream, which will eventually lead them to secondary target organs. Cancer cell invasion is facilitated by the activity of key enzymes involved in the degradation of the basement membrane. An important enzyme family implicated in tumor invasion is the collagenase family, which includes matrix metalloproteinase. Depending on the cancer type, the expression and activity of these enzymes can be influenced by the presence of IL-1β. For instance, IL-1β stimulation promotes the invasion of MCF-7, a non-metastatic breast cancer cell line, by inducing the expression of MMP9 in a dose-dependent manner [105]. In bladder cancer patients’ samples, IL-1β is highly expressed and induces the urokinase-type plasminogen activator receptor and MMP9 expression by activating the MAPK and NF-κB pathways, thus promoting bladder cancer cell invasion [106,107]. Furthermore, IL-1β works in concert with TGF-β to promote invasiveness in non-small cell lung cancer by inducing the expression of several MMPs (MMP-1, MMP-3, and MMP-10) [108,109]. Unsurprisingly, the knockdown of IL-1β and MMP-1 decreases the invasiveness of triple-negative breast cancer [110]. Aside from MMPs, IL-1β stimulation also increases the invasion of oral squamous carcinoma cell lines by inducing the expression of other proteases, such as ADAM9 and kallikrein 11 [111].

### 5.3. Angiogenesis

Angiogenesis is the generation of new vascular networks that aid the progression and survival of cells. For the successful establishment of metastases, angiogenesis is a crucial step needed for the dissemination of cancer cells. As discussed earlier, VEGF is a known pro-angiogenic factor. Its upregulation is implicated in several cancer types [110,111,112]. Voronov et al. reported a decrease in the formation of lung metastases and disruption in the recruitment of angiogenetic networks in B16 melanoma IL-1β knockout mice compared to wild-type mice [112,113]. They further corroborated their studies by demonstrating that blockade of IL-1β signaling significantly decreases tumor angiogenesis in C57B16 IL-1β knockout mice compared to wild-type and IL-1α knockout mice [112], highlighting the importance of IL-1β in promoting angiogenesis in cancer cells [112,113]. Leptin, a hormone mainly secreted by adipose tissue and involved in regulating body weight and pro-inflammatory response, can induce the expression of IL-1β [114,115], which upregulates downstream VEGF expression and signaling in breast cancer [116,117,118]. Conversely, other studies have reported that IL-1β can induce leptin expression during inflammation, suggesting an interplay between the these two molecules [119,120,121]. Lindahl et al. reported a decrease in the tumor burden and micro-vessel density of BALB/c nude mice that received IL-1Ra compared to the control group in breast cancer [122].

In addition to inducing angiogenesis, tumors are also capable of undergoing vasculogenic mimicry, a process that involves the formation of vessels lacking endothelial cells, functioning as a substitute perfusion network to promote their survival and invasiveness in hypoxic conditions. Vasculogenic mimicry has been reported in several cancer types, including breast and ovarian cancers and melanoma [123,124,125,126,127,128,129,130,131,132]. Unsurprisingly, IL-1β has been implicated in the vasculogenic mimicry of aggressive tumors. For instance, stimulation of breast cancer cell lines with IL-1β increased the formation of vascular channels in both hypoxic and normoxic conditions [133].

### 5.4. Conditioning of the Metastatic Niche

The interplay between IL-1β and chemokines is critical for recruiting and activating immune cells during inflammation. While different chemokines can modify the production and activity of IL-1β, IL-1β can also affect how chemokines are both expressed and function. In this context, the expression of a chemokine receptor involved in metastasis (CXCR4) can be upregulated by IL-1β in tongue squamous cell carcinoma in a dose and time-dependent manner [134]. This upregulation of CXCR4 leads to increased ERK and Notch signaling, supporting cancer cells’ survival and metastasis [134]. Furthermore, culturing mesenchymal stem cells (MSCs) in a medium conditioned by a metastatic breast cancer cell line resulted in the secretion and upregulation of several chemokines, as compared to a culture medium conditioned by cancer cells lacking metastatic potential. Notably, this effect was reduced when MSCs were cultured in a medium conditioned by metastatic cells with silenced IL-1β expression, hinting to a potential role of IL-1β in spawning a pro-metastatic microenvironment via the upregulation of chemokine secretion by stromal cells [58].

This concept seems particularly relevant for the skeletal metastatic niche. For instance, cytokines and chemokines secreted by the osteoblasts can be used by breast cancer cells to aid their survival in the bone microenvironment [135]. Conversely, culturing the non-invasive breast cancer cell line MCF-7 with an osteoblast-conditioned medium increased both cytokine secretion and the migration rate of the cancer cells [136]. Zhao et al. reported that N-acetyltransferase-1 promotes osteolytic bone metastasis in luminal breast cancer via IL-1β signaling and NF-κB activation, [137]. Additionally, co-culture of breast cancer cell lines and human femur tissue derived from patients undergoing hip replacement surgery induced the upregulation of IL-1β and leptin and promoted the colonization of cancer cells within the bone marrow adipose tissue [138]. Studies by Nutter et al. identified IL-1β as a gene upregulated in MDAP-IV, a bone metastatic breast cancer cell line generated in their lab compared to its parental MDA-MB-231 counterpart [59]. Interestingly, they reported no difference in the proliferation rate and invasiveness between the two cell lines [59], supporting the widely accepted idea that the ability of tumor cells to colonize the skeleton strongly depends on extrinsic factors present in the metastatic niche [139].

Finally, chemokines can increase immune cells’ expression of IL-1Rs, making them more susceptible to IL-1β signaling. This could lead to a feedback loop where IL-1β stimulates the production of chemokines, enhancing the secretion and activity of IL-1β that can activate signaling pathways promoting cancer proliferation and metastasis [140].

## 6. Prostate Cancer: Clinical Course and Treatment

### 6.1. Localized Disease

Of all male cancers, the global incidence of prostate cancer is exceedingly high (13.5%). It is the second most frequently diagnosed cancer in men after lung cancer (14.5%) [141,142]. Prostate cancer risk increases with age, with >70% of newly diagnosed men being >65 years of age [143]. Although its incidence is high, prostate cancer mortality is relatively low. Prostate cancer only accounts for 6.7% of all cancer deaths in men, compared with 22.0% for lung cancer [141]. This is the result of the excellent prognosis for locally confined disease, with survival being as high as 99% over 10 years if diagnosed at an early stage [144]. In men 55–69 years of age with no family history of cancer or <55 years of age with a family history of any cancer [142,145], prostate-serum antigen (PSA) levels higher than 4.0 ng/mL and/or palpable prostate enlargement upon digital rectal exam (DRE) raise the suspicion of prostate cancer. However, prostatic enlargement is a normal part of the male aging process, so clinicians must differentiate between prostate cancer and benign prostatic hyperplasia (BPH) [146]. In fact, the positive predictive values (PPV) for PSA and DRE are only ~25.1% and ~17.8%, respectively [147]. Thus, a definitive diagnosis of prostate cancer requires biopsy and histopathological verification [146].

For localized prostate tumors, the aggressiveness of treatment is selected based on the risk of malignancy. Active surveillance (monitoring PSA increases over time often combined with multiparametric MRI), radiotherapy, and/or radical prostatectomy are currently considered depending on individual risk factors [142]. After radiotherapy and/or radical prostatectomy, PSA levels are monitored for biochemical recurrence (BCR), which indicates residual disease. BCR eventually occurs in 20–40% of patients after radical prostatectomy and/or radiotherapy [148]. If BCR occurs without detectable metastases, salvage radiotherapy (SRT) can be used to irradiate the former prostatic tumor bed and prevent metastatic progression. SRT achieves a 75% risk reduction for systemic progression [149] and an 88% chance of remaining progression free after 5 years [150,151]. However, BCR might also indicate the existence of metastatic deposits, which at early stages may escape conventional diagnostic imaging (i.e., MRI, CT, and bone scintigraphy). Newer, more sensitive methods such as PSMA-PET have a higher likelihood of radiologically detecting micrometastases in patients with BCR [152].

### 6.2. Metastatic Disease

Treatment is largely curative for locally confined disease. Over a 10-year period, metastatic progression only occurs in ~4% of patients receiving treatments for localized prostate cancer [153]. On the other hand, de novo metastatic disease, in which secondary tumors exists at the time of diagnosis, is detected in 5% of newly diagnosed patients [142]. Metastatic progression necessitates systemic treatment. Androgens are necessary growth factors for prostate tissue, and this dependency can be exploited by ADT. Since the first piece of evidence that androgen deprivation hampered prostate cancer growth emerged [154], ADT was achieved by surgical orchiectomy (bilateral resection of the testes). However, this approach has largely been replaced by chemical castration via LHRH agonists (negative feedback) or LHRH antagonists (direct inhibition) [155,156]. The disease initially responds well to ADT and at this stage is termed metastatic castration-sensitive prostate cancer (mCSPC). However, mCSPC patients invariably progress, thus transitioning into metastatic castration-resistant prostate cancer (mCRPC) [157]. The emergence of therapy resistance in mCSPC can be etiologically explained by two major factors. First, although ADT suppresses testosterone synthesis via the gonadal pathway, residual adrenal testosterone synthesis remains [158]. This can be addressed by the adoption of nonsteroidal androgen receptor inhibitors (ARIs) such as enzalutamide, apalutamide, and darolutamide, which directly antagonize the AR [159].

Steroidal anti-androgens (SAAs) such as abiraterone act further upstream, antagonizing the CYP17A1 enzyme which is required in the testosterone synthesis pathway [142]. Metastatic progression despite ADT + ARIs/SAAs occurs due to a range of mechanisms and events, including AR activating mutations and amplification [142,160,161] and/or its downregulation, and the development of AR-independent mechanisms to support tumor growth and survival [162,163,164]. At this late clinical stage, nonspecific chemotherapies have largely remained as a first-line treatment [155]. Unbridled cellular hyperproliferation, a hallmark of cancer, can be targeted via microtubule-stabilizing taxanes such as docetaxel and cabazitaxel. These drugs disrupt mitotic spindle dynamics and trigger apoptosis upon mitotic arrest. mCRPC patients with disease progression after taxane treatment have several second-line therapeutic options, with differing clinical profiles dictating treatment of choice. Since prostate cancer preferentially metastasizes to the skeleton, radium-223 (a bone-targeting, α-particle-emitting radionuclide) is often administered to patients experiencing symptoms from osseous metastases [165]. Germline and somatic genetic testing can be used to identify patients with specific DNA damage repair (DDR) deficiencies. Patients with homologous recombination (HR) deficiencies (frequently associated with loss of function mutations in *BRCA1*, *BRCA2*, and *ATM*) respond well to poly ADP ribose polymerase inhibitors (PARPi) such as Olaparib. Patients with mismatch repair (MMR) deficiencies resulting in high microsatellite instability (MSI-H) respond well to anti-PD1 treatments such as Keytruda [155]. Prostate cancer’s characteristic surface expression profile can also be exploited as a treatment target. Sipuleucel-T is an autologous dendritic cell vaccine which immunizes against the prostatic acid phosphatase (PAP) epitope and triggers immune destruction via T cell recognition. Finally, Lutetium-177 (^177^Lu, a β-particle-emitting radionuclide) can be conjugated to the prostate-specific membrane antigen (PSMA) to form the targeted radiopharmaceutical ^177^Lu-PSMA [142].

Despite these treatment regimens, which use several biological targeting strategies, the prognosis for mCRPC patients remains grim. The median overall survival (OS) after disease progression in either mCSPC or mCRPC setting is only 23 months and 17 months, respectively [166]. Given the high prevalence of prostate neoplasia and the high mortality rate upon systemic progression, new, more efficacious treatment options for metastatic prostate cancer are of paramount importance to the field of medical oncology.

In addition to inducing angiogenesis, tumors are also capable of undergoing vasculogenic mimicry, a process that involves the formation of vessels lacking endothelial cells, functioning as a substitute perfusion network to promote their survival and invasiveness in hypoxic conditions. Vasculogenic mimicry has been reported in several cancer types, including breast and ovarian cancers and NSCLC [123,124,125,126,127,128,129,130,131,132]. Unsurprisingly, IL-1β has been implicated in the vasculogenic mimicry of aggressive tumors. For instance, stimulation of breast cancer cell lines with IL-1β increased the formation of vascular channels in both hypoxic and normoxic conditions [133].

## 7. Tumor-Derived IL-1β in Bone Metastatic Prostate Cancer

The role of IL-1β in tumor development, survival, and metastasis has been well established, and some examples have been discussed earlier in this review. The skeleton is a leading secondary site for seeding and colonization of cancer cells, particularly in prostate cancer. Bone metastases are responsible for relevant cancer-related morbidity and are associated with a high mortality rate [167,168,169,170,171,172]. The bone microenvironment consists of several autochthonous cell types, including mesenchymal cells, osteoblasts, osteoclasts, and adipocytes, in addition to cells with hematopoietic functions [173,174]. In healthy patients, the bone’s integrity and function depends on the balance between bone formation and resorption (referred to as bone remodeling) by osteoblasts and osteoclasts, respectively. However, in bone metastasis, dysregulation of the bone remodeling processes results in osteoblasts and osteoclasts repeatedly engaged in a vicious cycle, in which the release of growth factors from the mineralized matrix promotes the colonization of cancer cells and expansion of the tumor mass at the expense of the bone tissue [175,176]. In >80% of patients with advanced prostate cancer, the skeleton is the only site of metastasis, whereas only ~10% of patients harbor secondary tumors in both the skeleton and soft tissues [177,178,179]. These data indicate that the bone is the first site of metastatic spread of prostate cancer cells, which is similar to what is observed in patients with advanced breast cancer [180]. For both types of tumors, dissemination to soft tissues dramatically worsens cancer-related survival [178], and there is evidence that the bone microenvironment allows for the acquisition of specific traits for the further dissemination of cancer cells to soft tissue organs such as liver, lungs, and brain [181,182]. Therefore, treatments that effectively counteract skeletal colonization will inevitably not only improve quality of life but also significantly extend the life expectancy of prostate cancer patients.

Decades of research have highlighted the contribution of IL-1β to osteoclasts’ differentiation and activity in vitro and in vivo [183,184,185,186,187,188]. Osteoclasts are responsible for osteolysis and bone resorption, which are major contributors to the complications observed in patients with bone metastasis [175,189]. Mechanisms by which IL-1β promotes bone remodeling via osteoclast activities include multinucleation of osteoclasts independent of osteoblast activity and upregulation of RANKL expression via TNF-α activity [107,190,191]. Moreover, IL-1β was identified as an essential factor that supports the activity of the osteoclast-activating factor [185].

**Figure 1:**
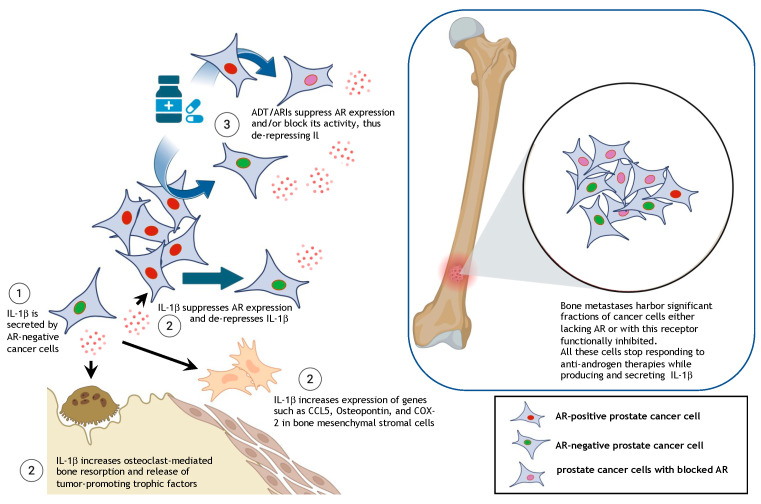
IL-1β secretion in the prostate cancer bone metastatic niche. (**1**) Tumor cells lacking the AR secrete IL-1β, which causes (**2**) the release of soluble trophic factors via the activation of bone matrix resorption by osteoclasts and induces changes in the transcriptomic profile of bone mesenchymal stromal cells. (**3**) ADT/ARIs causes both an increase in AR-negative cancer cells and the inhibition of AR signaling and consequent de-repression and secretion of IL-1β from AR-positive tumor cells. These events lead to metastatic tumors harboring cancer cells heterogenous for AR expression or activity, but homogeneous for IL-1β production. Generated with Biorender.

Our group was the first to establish a functional link between IL-1β expression and the bone metastatic potential of prostate cancer cells in pre-clinical models [62]. This finding was followed by a study by Schulze et al., reporting the induction of chemokine expression in osteoblasts exposed to culture medium conditioned by PC3 human prostate cancer cells and containing IL-1β [192]. In further studies we reported that the IL-1R antagonist anakinra dramatically reduced the number and size of skeletal tumors from PC3-ML cells grafted in the systemic blood circulation of mice. Consistently, we also found that PC3-ML cells were unable to colonize the skeleton of mice knocked out for IL-1R [63]. These findings indicate that the tumor-associated bone stroma is engaged via activation of IL-1R and is likely responsible for spawning a tumor-permissive metastatic niche. This concept was corroborated by the Nanostring^®^ profiling of tumor-associated stroma, showing the altered expression of 30 genes, including the upregulation of the chemokine CCL5, the trophic factor osteopontin, and the inflammation-related COX-2 enzyme (Figure 1), among others [63]. Notably, the conditioning of the bone metastatic niche by IL-1β secreted by PC3-ML cells supported the survival and growth of prostate cancer cells such as LNCaP cells (growing within the same skeletal tumors) that otherwise would have failed to colonize the bone. This provided evidence for metastatic cooperation among different prostate cancer phenotypes via IL-1β signaling [63], which in addition to recruiting the bone stroma could also directly affect cancer cells through autocrine and paracrine stimulation [193,194,195,196]. In fact, direct stimulation of IL-1R on prostate cancer cells suppresses AR expression [195], thus potentially instigating further secretion of this cytokine in the surrounding tumor-associated stroma (Figure 1). Additional studies reported an IL-1β-induced inflammatory phenotype of bone marrow adipocytes by PC3 cells directly grafted in mouse bone, which reduced the cytotoxic effects of docetaxel via lipolysis-dependent mechanisms [64].

Thus, these findings offer evidence for multiple roles exerted by IL-1β in promoting metastatic prostate cancer, similar to what was reported in bone tropic breast cancer [60,197,198,199], and provide the bedrock for targeting IL-1β signaling in both tumors as a novel treatment strategy.

## 8. The Androgen Receptor Represses IL-1β Transcription

The AR signaling axis and transcriptional activities drive the proliferation of the hormone-sensitive form of prostate cancer [200]. In its inactive state, the AR is located in the cytosol and bound to heat shock proteins. Upon binding testosterone or dihydrotestosterone in the cytosol, the AR undergoes a conformational change, separating from the heat shock proteins and exposing a nuclear localization signal. This is required for its translocation to the nucleus as a homodimer and binding to androgen-responsive elements (AREs) within target genes to repress or activate their transcription [201,202].

As mentioned above, PC3-ML human prostate cancer cells express and secrete IL-1β [62]. These cells lack AR expression, thus reproducing a phenotype detected in 23% of metastatic patients and 21% of metastatic tumors. Furthermore, considering prostate cancer cells with neuroendocrine features, AR-negative phenotypes are cumulatively found in 36% of patients and 31% of metastatic tumors [203], a strong indication of the importance of cancer cells lacking the AR in advanced disease stages [162,163,164,204].

In contrast, AR-positive prostate cancer cells fail to express the cytokine. We confirmed this AR/IL-1β inverse correlation in both cell lines and patients by immunohistochemistry and RNA sequencing [63,205]. Mechanistically, we discovered that the AR represses IL-1β transcription by binding to ARE half-sites on the promoter region of the gene [205]. This is particularly relevant, not only to understand the secretion of IL-1β by AR-negative phenotypes, but also in light of the inhibition of AR activity by ADT and ARIs commonly used in therapy [203,206,207]. These also de-repress IL-1β in cancer cells expressing the AR, which are otherwise unable to produce this cytokine.

Taken together, these findings have compelling clinical significance. In a highly plausible model, AR-negative cancer cells inhabiting metastatic tumors secrete IL-1β and the signaling by this cytokine elicits the production of tumor-promoting factors by the surrounding stromal cells via IL-1R stimulation. These effects are compounded by the adoption of treatment regimens such as ADT and/or ARIs (Figure 1).

Notably, an inactive AR signaling does not unleash IL-1β expression in all prostate cancer phenotypes. By analyzing whole-genome bisulfite sequencing data from one-hundred metastatic patients [208], we identified 346 CpG sites spanning the IL-1β promoter and gene body on chromosome 2, of which 34 showed significant differences in methylation when comparing patients with similarly low AR activity but having high vs. low expression of IL-1β. [205]. Our findings are in line with other reports of DNA methylation regulating IL-1β gene expression [209,210,211]. These findings present important implications for therapeutic strategies aimed to block IL-1β signaling, which will be discussed in the next paragraph.

## 9. Rationale for Combining ADT/ARIs with Inhibitors of IL-1β Signaling

Currently, there are three FDA-approved agents for the blockade of the IL-1β signaling pathway [212]. Each agent achieves IL-1β blockade through a unique mechanism. Anakinra (Kineret) first received FDA approval in 2001 for the treatment of rheumatoid arthritis (RA). It is a recombinant form of the human IL-1R antagonist, which works by competitively inhibiting the binding of both IL-1α and IL-1β to IL-1R [213]. Canakinumab (Ilaris) first received FDA approval in 2009 for the treatment of cryopyrin-associated periodic syndrome (CAPS). It is a fully human monoclonal antibody that selectively binds to and neutralizes IL-1β [214]. Finally, rilonacept (Arcalyst) first received FDA approval in 2008 for the treatment of CAPS. It is a fusion protein consisting of the extracellular domains of IL-1R1 and the IL-1 receptor accessory protein (IL-1RAcP) linked to the Fc portion of human IgG1. This structure allows rilonacept to act as a soluble decoy receptor that binds to and neutralizes both IL-1α and IL-1β [215].

Numerous clinical trials have been conducted to expand the indications of these three IL-1β blocking agents. In 2017, the canakinumab anti-inflammatory thrombosis outcomes study (CANTOS) was completed. CANTOS was a randomized, double-blind, placebo-controlled trial conducted with 10,061 patients who were stable after myocardial infarction (MI). The study explored whether IL-1β blockade via canakinumab treatment could prevent atherosclerotic disease (i.e., nonfatal MI, nonfatal stroke, or cardiovascular death) in post-MI patients who exist in a proinflammatory state (defined by high-sensitivity C-reactive protein (hsCRP) levels ≥ 2 mg/L). The primary objective of the CANTOS trial was successful. The study found that canakinumab at a dose of 150 mg every 3 months led to a significantly lower rate of recurrent atherosclerotic disease than placebo [216]. However, CANTOS also unexpectedly shed light on the potential of canakinumab as a therapeutic in the oncology space. To assess canakinumab’s adverse effect profile, all study participants were followed prospectively for incident medical events for 3–5 years. The study found that canakinumab lowered the hazard ratio (HR) of fatal cancer incidence (HR = 0.45, N = 115, *p* = 0.0158). This effect was most pronounced in fatal lung cancer incidence (HR = 0.15, N = 39, *p* = 0.0026) [217,218].

The agents listed above represent an arsenal of therapeutics that could be repositioned to treat additional clinical indications, including neoplastic diseases for which a tumor-promoting role of IL-1β signaling is identified. Prostate cancer is one of these diseases, and particularly in patients with its advanced form, the lack of AR expression—either constitutive or induced in AR-positive cells by paracrine IL-1β signaling—might be compounded by the functional inactivation of the AR by ADT/ARIs.

**Figure 2:**
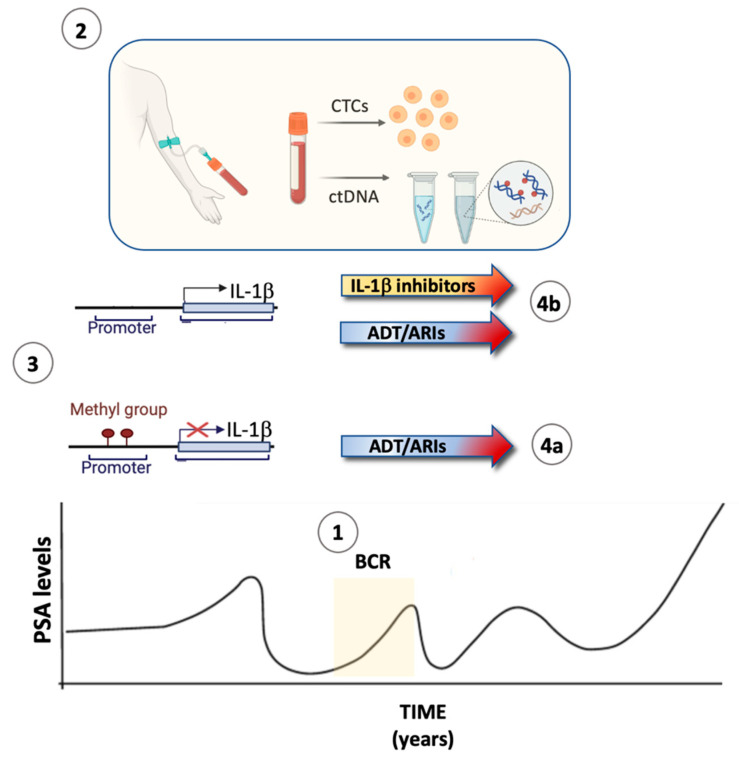
Proposed biomarker-informed treatment of prostate cancer patients. Following successful treatment of locally confined prostate cancer by surgery and/or radiations, the plasma levels of prostate-specific antigens (PSA) decrease to undetectable levels. (**1**) A resurgence in PSA levels is defined as biochemical recurrence (BCR) and indicates the likelihood of minimal residual disease. Currently, BCR is treated with ADT, often combined with an ARI. We propose to (**2**) test patients for AR/IL-1β expression in circulating tumor cells (CTCs) collected from peripheral blood and for methylation of the IL-1β gene locus by collecting circulating DNA (ctDNA). Depending on the (**3**) methylation status of the IL-1β gene locus, patients with a methylated locus and therefore negative IL-1β expression would be treated with ADT alone (**4a**). Patients carrying an unmethylated IL-1β locus would be candidates for a combinatorial approach of ADT/ARIs plus inhibitors of IL-1β signaling (**4b**). Generated with Biorender.

To specifically address this scenario, a plausible clinical approach would be to collect circulating tumor cells (CTCs) before and after starting ADT (with or without combination with ARIs) and assess AR status [219] and IL-1β expression. In alternative or in combination, the methylation status of the IL-1β locus could be assessed in CTCs or by examining circulating DNA. This strategy would preemptively identify patients lacking methylation and up-regulating IL-1β, similar to what is currently implemented for other tumors and biomarkers [220,221,222,223] (Figure 2).

## 10. Conclusions

The evidence provided above strongly supports a crucial role of IL-1β in promoting tumor growth and progression. This applies when IL-1β is secreted by immune cells and might be even more relevant when the source of IL-1β is the cancer cells themselves. For instance, for tumors routinely identified as *immunologically cold* [224]—harboring either few or exhausted immune cells—IL-1β is likely to exert additional effects through the activation of signaling pathways in bystander normal cells inhabiting the tumor-associated tissue microenvironment. This seems to be particularly relevant for the metastatic niche of target organs, and this review has provided several examples of this paradigm. A particularly striking scenario is the bone metastatic niche in which prostate cancer cells, either lacking the AR or with this receptor inactivated by therapy, produce and secrete IL-1β, which is normally repressed by the binding of the AR to at least one ARE half-site near the IL-1β promoter. Pre-clinical studies have shown how this can promote tumor progression and should be clearing the way for clinically assessing the therapeutic value of inhibiting IL-1β signaling in prostate cancer. Thus, the adoption of biomarker-informed treatments blocking IL-1β seems bound to significantly improve the clinical outcome of prostate cancer and other tumors, as shown by recent clinical trials with metastatic breast cancer patients [225].
